# Treatment of recurrent aphthous stomatitis. A literature review

**DOI:** 10.4317/jced.51401

**Published:** 2014-04-01

**Authors:** Irene Belenguer-Guallar, Yolanda Jiménez-Soriano, Ariadna Claramunt-Lozano

**Affiliations:** 1 Degree in Dental Surgery. Master in Oral Medicine and Surgery; 2 Assistant Professor. University of Valencia. Valencia, Spain

## Abstract

Recurrent aphthous stomatitis (RAS) is the most common chronic disease of the oral cavity, affecting 5-25% of the population. The underlying etiology remains unclear, and no curative treatment is available. The present review examines the existing treatments for RAS with the purpose of answering a number of questions: How should these patients be treated in the dental clinic? What topical drugs are available and when should they be used? What systemic drugs are available and when should they be used? A literature search was made of the PubMed, Cochrane and Scopus databases, limited to articles published between 2008-2012, with scientific levels of evidence 1 and 2 (metaanalyses, systematic reviews, phase I and II randomized clinical trials, cohort studies and case-control studies), and conducted in humans. The results obtained indicate that the management of RAS should be based on identification and control of the possible predisposing factors, with the exclusion of possible underlying systemic causes, and the use of a detailed clinical history along with complementary procedures such as laboratory tests, where required. Only in the case of continuous outbreaks and symptoms should drug treatment be prescribed, with the initial application of local treatments in all cases. A broad range of topical medications are available, including antiseptics (chlorhexidine), antiinflammatory drugs (amlexanox), antibiotics (tetracyclines) and corticosteroids (triamcinolone acetonide). In patients with constant and aggressive outbreaks (major aphthae), pain is intense and topical treatment is unable to afford symptoms relief. Systemic therapy is indicated in such situations, in the form of corticosteroids (prednisone) or thalidomide, among other drugs.

** Key words:**Recurrent aphthous stomatitis, treatment, clinical management.

## Introduction

Recurrent aphthous stomatitis (RAS) is characterized by the appearance of initially necrotic ulcers, with well defined limits surrounded by an erythematous halo. The lesions are located on the oral mucosa, but are infrequent on the gums (1,2). The disease manifests in the form of outbreaks, with a chronic and self-limiting course in most cases (3,4). RAS is the most frequent chronic disease of the oral cavity, affecting 5-25% of the population (3-6). It is more common in patients between 10-40 years of age, and predominantly affects women and individuals of higher socioeconomic levels (1,3,7).

The underlying etiology is not clear, though a series of factors are known to predispose to the appearance of oral aphthae, including genetic factors, food allergens, local trauma, endocrine alterations (menstrual cycle), stress and anxiety, smoking cessation, certain chemical products and microbial agents (2-5,8,9). Immune alterations have been observed, beginning with an unknown antigenic stimulation of the keratinocytes, and resulting in the activation of T lymphocytes, cytokine secretion (including tumor necrosis factor-alpha (TNF-α), and leukocyte chemotaxis. TNF-α is believed to play an important role in the development of new RAS lesions, and has been found to be increased 2- to 5-fold in the saliva of affected patients (10). Changes have also been reported in elements of the salivary defense system, such as the enzyme superoxide dismutase (SOD), which participates in the inflammatory response of RAS (11). An increase is moreover observed in the expression of vascular and keratinocyte adhesion molecules. This gives rise to the accumulation of lymphocytes and lymphocyte infiltration of the epithelium, with the induction of ulcer formation (3,4,12). On the other hand, many systemic diseases are known to be associated with oral aphthae, including Behçet’s syndrome, hematological disorders, vitamin deficiencies, gastrointestinal diseases, cyclic neutropenia, Reiter syndrome, Magic syndrome, PFAPA (periodic fever, aphthous pharyngitis and cervical adenopathy), Sweet syndrome and immune deficiencies (1,13).

As regards the clinical manifestations, the basic lesion is a recurrent, painful, rounded or oval ulcer with a necrotic base. Three clinical subtypes of RAS have been established according to the magnitude, number and duration of the outbreaks (12):

• Minor RAS: This is the most common presentation of the disease, representing 70-85% of all cases. It manifests as small rounded or oval lesions covered by a grayish-white pseudomembrane and surrounded by an erythematous halo. Each minor RAS episode usually involves the appearance of 1-5 ulcers measuring under 1 cm in diameter. These episodes are self-limiting and resolve within 4-14 days without leaving scars (3-5,7,9).

• Major RAS: This is the most severe presentation of the disease, representing 10% of all cases. In this subtype the ulcers measure over 1 cm in size and tend to appear on the lips, soft palate and pharynx. The lesions persist for over 6 weeks and can leave scars.

• Herpetiform RAS: This subtype accounts for 1-10% of all cases and is characterized by recurrent outbreaks of small, deep and painful ulcers. Up to 100 aphthae can develop simultaneously, measuring 2-3 mm in size, though they tend to merge to form larger ulcerations with an irregular contour. This presentation is more often seen in women and in patients of older age, in contrast to the other two clinical subtypes of the disease (3,4,9,12).

The diagnosis of RAS is based on the patient anamnesis and clinical manifestations. There is no specific diagnostic test, though it is essential to discard possible underlying systemic causes – particularly in the case of adults who suffer sudden outbreaks of RAS. It is advisable to request a complete series of laboratory tests, including a complete blood count, and evaluations of iron, vitamin B12 and folic acid. A biopsy of the lesions is only recommended in the case of diagnostic uncertainty, since the findings only indicate a simple nonspecific inflammatory lesion (3,4,12).

Since the cause of the disease is not known, many drugs have been evaluated in an attempt to palliate the symptoms. Treatment used is multifocal and varies according to the predisposing factors. In all cases management is symptomatic, and seeks to reduce inflammation of the aphthae and afford pain relief by administering topical or systemic treatments (1,2).

The present literature review examines the existing treatments for RAS with the purpose of answering a number of questions: How should these patients be treated in the dental clinic? What topical drugs are available and when should they be used? What systemic drugs are available and when should they be used?.

## Material and Methods

A literature search of the PubMed, Cochrane and Scopus databases was made using the key words recurrent aphthous stomatitis, treatment and clinical management, combined and related by means of the boolean operator AND. The search was limited to articles published between 2008-2012, with scientific levels of evidence 1 and 2 (metaanalyses, systematic reviews, phase I and II randomized clinical trials, cohort studies and case-control studies), and conducted in humans. A total of 33 articles were identified: one metaanalysis, one cohort study, 3 systematic reviews and 24 phase I and II randomized clinical trials. We also included four literature reviews, due to their interest and recent publication.

## Results and Discussion

The literature describes different approaches to the management of RAS. As a first consideration, we will describe the recommendations on how these patients should be treated in the dental clinic.

- Initial clinical evaluation and non-pharmacological treatment

A first requirement is a full and detailed clinical history (3,4). Some authors recommend complementary measures such as a complete blood test including red cell count, folic acid, ferritin and vitamin B12, with the purpose of discarding possible underlying systemic causes (vitamin deficiencies, gastrointestinal disease, Behçet’s syndrome, immune deficiencies) – particularly in the case of adults who suffer sudden outbreaks of RAS, in patients with major aphthae, or when there are also lesions in other parts of the body (3,4,9). Due to the relationship between RAS and vitamin deficiencies, some authors such as Volkov *et al*. (8) have reported that treatment with vitamin B12, apart from being simple, inexpensive and of low risk, proves effective in application to RAS, even independently of the serum vitamin B12 levels of the patient. Other investigators such as Baccaglini *et al*. have reported similar results (1). Treatment with 2 g of vitamin C a day during three months has also been shown to be effective, as in the study published by Yasui *et al*. (14), who documented a decrease of at least 50% in the frequency of RAS outbreaks with such therapy. However, other authors consider that daily multivitamin supplements are unable to reduce either the number or the duration of RAS outbreaks, and therefore consider that physicians should not recommend such supplements on a routine basis as preventive treatment (13).

The treatment prescribed should be conditioned to the severity of the disease (pain), the medical history of the patient, the frequency of the outbreaks, and patient tolerance of the medication (1,3).

In order to facilitate definition of the best treatment option, the patients can be classified according to their clinical characteristics as follows:

• Type A: Brief episodes occurring only a few times during the year, and characterized by tolerable pain levels. Predisposing factors should be identified and controlled (e.g., avoiding local trauma, using a soft toothbrush, providing brushing instructions). It is advisable to question the patient about his or her eating habits, in order to evaluate possible associations between the disease outbreaks and certain foods. In this context, it is generally advisable to avoid hard foods (e.g., hard toasted bread), all types of nuts (walnuts, hazelnuts, etc.), chocolate, acid beverages or foods (fruit or citrus juices, tomato), salty foods, very spicy food (pepper, curry) and alcoholic and carbonated beverages.

• Type B: Episodes develop on a monthly basis, lasting 3-10 days, and the pain causes the patient to modify habits of hygiene and diet. If a predisposing factor is identified (trauma, stress, diet, hygiene, etc.), it should be commented with the patient and controlled. It is important to question about prodromic manifestations (itching or swelling), in order to provide topical treatment when these occur.

• Type C: The episodes are very painful, with chronic aphthae. Some lesions develop while others heal, and the patient does not respond to topical treatment. In such cases systemic therapy is indicated (1,3,4,12).

- Local pharmacological treatment

Treatment should always start with topical medication ( Table 1). The first line treatment options comprise antiseptics and antiinflammatory drugs/analgesics such as 0.2% chlorhexidine in rinses or gel, three times a day (without swallowing), for as long as the lesions persist. Triclosan can also be used in gel or rinse format three times a day (without swallowing), for as long as the lesions persist, and affords antiinflammatory, antiseptic and analgesic effects. In turn, topical 3% diclofenac with 2.5% hyaluronic acid can be applied to lessen the pain (12). There have also been reports of the use of oral rinses with benzidamine hydrochloride, which offers temporary pain relief (1,12).

**Table 1:**
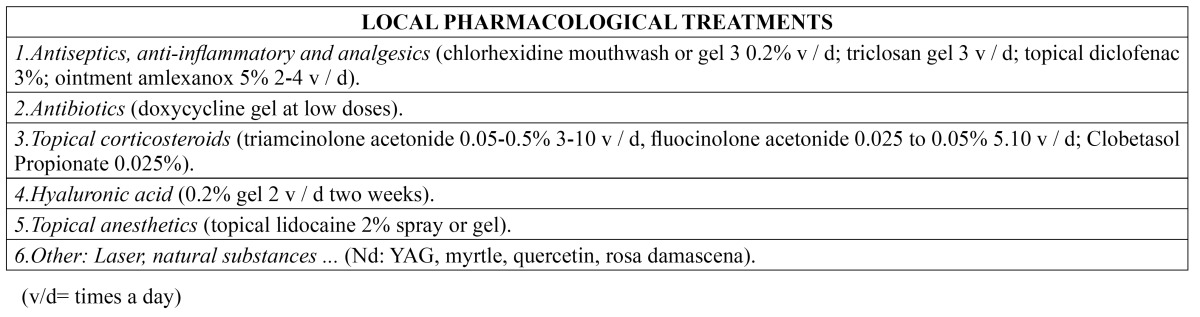
Local pharmacological treatments.

Amlexanox is a widely studied drug that offers short-term efficacy, particularly when used in the prodromic (early symptoms) phase. Its mechanism of action is not known, though it is a topical agent with established antiinflammatory and antiallergic properties (1). It is usually supplied in the form of an ointment at a concentration of 5%, and is applied 2-4 times a day. The drug has been shown to be effective in accelerating the healing of aphthae and in lessening the pain, erythema and size of the lesions (7). It has also been studied in other presentations such as oral patches, tablets or adhesive films. Meng *et al*. (7) observed no significant differences in efficacy between the application of amlexanox in patch or tablet form, though patients appear to prefer the former presentation.

Topical antibiotics such as tetracyclines and their derivatives (doxycycline and minocycline), in gel or rinse format, have also been found to lessen the pain and outbreaks of RAS. These drugs act through the local inhibition of collagenases and metalloproteinases (MPs) that form part of the inflammatory response and contribute to tissue destruction and ulcer formation, and moreover exert immune modulating effects (1). Of the commercially available tetracyclines, doxycycline has shown the best inhibition of MPs (15). The administration of fixed-dose doxycycline in mucoadhesive gel format has been shown to be effective in treating RAS. Other authors recommend its application at a dose of 100 mg in 10 ml of water, performing rinses for 2-3 minutes (without swallowing), four times a day during three days (12). The topical use of tetracyclines and retinoic acid also exerts an antiinflammatory effect, in addition to the known antibiotic action (16).

The most widely used drugs in immune-mediated oral mucosal diseases are the topical corticosteroids. The aim of such treatment is to eliminate the symptoms, thereby allowing the patient to eat, speak and perform normal oral hygiene, since topical corticosteroids reduce or even suppress the pain and shorten the aphthae healing time (6). In patients with RAS, the indicated drugs are triamcinolone acetonide, fluocinolone acetonide or clobetasol propionate, in order of lesser to greater potency, according to the severity of the lesions. These three drugs can be administered as a pomade in orabase when the lesions are of a localized nature, or in rinse format when the lesions are diffuse or very numerous. Triamcinolone acetonide is used at concentrations ranging from 0.05-0.5%, applied 3-10 times a day during 3-5 minutes. It is particularly indicated in patients with small and mild erosive lesions. Some authors consider the most effective concentration to be 0.1% (6,12,16). In order to facilitate healing, it is advisable to apply the medication directly onto the lesions, keeping it in direct contact for as long as possible, and taking care not to eat or drink during 20 minutes after application, or touch the treated zone. If the corticosteroid is administered as an oral rinse, it should be used for the indicated period of time, without swallowing the product. On the other hand, fluocinolone acetonide at a concentration of 0.025-0.05%, applied 5-10 times a day during 3-5 minutes, affords medium to high potency, and is widely used in patients with more aggressive lesions. Lastly, 0.025% clobetasol propionate is the most potent topical corticosteroid, and is therefore reserved for moderate or severe disease presentations. In this context, it is regarded as an alternative prior to the prescription of systemic therapy (6,12).

Another evaluated topical corticosteroid is dexamethasone. Liu *et al*. (17) investigated the efficacy and safety of dexamethasone pomade in treating RAS. They evaluated the size of the aphthae and their duration, as well as the intensity of pain, and concluded that the pomade is effective and safe when used in such situations. Al-Na´mah *et al*. (18) in turn compared a dexamethasone pomade with the commonly used triamcinolone acetonide formulation in orabase, and found both products to be equally effective in treating RAS.

Other topical treatments that have been used in RAS are 0.2% hyaluronic acid in gel formulation, applied twice a day during two weeks (19); topical anesthetics such as 2% lidocaine (as a spray or gel); adhesive toothpaste containing polydocanol; or benzocaine tablets (3,12). In turn, the Nd:YAG laser has been found to afford immediate pain relief and faster healing, and is well tolerated by patients with RAS, since it is a brief form of treatment, results in lesser pain after application, and has few side effects (20). Other treatments include natural substances such as myrtle (*Myrtus communis*), a bush from northern Iran that possesses blood glucose-lowering, antibacterial, analgesic and antioxidant properties, thus suggesting potential usefulness in application to diseases characterized by inflammation and allergy (21); quercetin, a flavonol found in fruits and vegetables, with antioxidant properties and which may prove useful in shortening aphthae healing time when applied as daily topical treatment (22); bioadhesive patches containing licorice hydrogel, which reduce the diameter of the inflammatory halo and the necrotic center of the aphthae, and the pain they produce (23); or oral rinses containing an aqueous extract of Damask rose, which possesses antiinflammatory and antinociceptive properties (24).

- Systemic pharmacological treatment

The outbreaks of RAS are normally resolved with topical treatments, though in some cases these measures prove insufficient because of the severity of the lesions or for unknown reasons. This is when second line therapy with systemic drug substances is indicated ( Table 2).

**Table 2:**
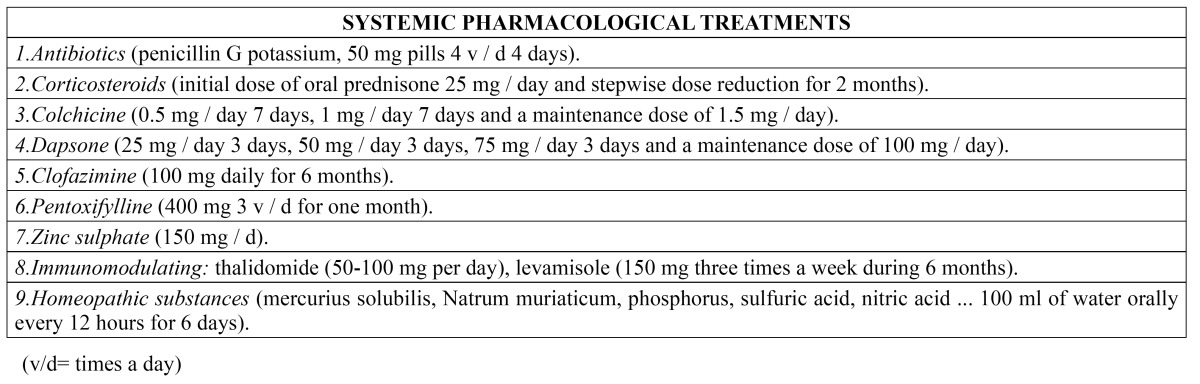
Systemic pharmacological treatments.

Studies have been made of systemic antibiotics such as potassium penicillin G in 50 mg tablets administered four times a day during four days, which help reduce the size of the ulcers and lessen the pain (5). The oral antipoliomyelitic vaccine has also been found to significantly reduce the duration of the aphthae, the frequency of outbreaks, and their severity (25).

The most effective treatments include corticosteroids and immunosuppressors. Pentoxifylline, colchicine, dapsone and thalidomide have also been used, but require caution because of possible adverse effects. These treatments are essentially palliative, since none of them have been able to secure permanent disease remission (26).

Corticosteroids are the first choice systemic treatment. They are usually used as rescue therapy in patients with acute severe RAS outbreaks (27). Oral prednisone has been used at a starting dose of 25 mg/day, followed by stepwise dose reduction, during two months, with disappearance of the pain and reepithelization of the lesions in the first month of therapy (26). The drug can produce long-term adverse effects; as a result, its efficacy has been compared with that of other drugs, in search of an alternative treatment. In this context, Femiano *et al*. (2) compared the efficacy of prednisone prescribed at a dose of 25 mg/day via the oral route during 15 days, 12.5 mg/day during 15 days, 6.25 mg/day during 15 days, and then 6.25 mg on alternate days during 15 days, in comparison with montelukast (a leukotriene receptor antagonist used as an antiasthma drug) 10 mg via the oral route each night, followed by administration on alternate days during the second month. The authors found both treatment modalities to be effective in reducing the number of lesions, affording pain relief and accelerating healing of the ulcers. As regards adverse effects, montelukast was found to be safer, and therefore should be taken into account as an option when systemic corticosteroids are contraindicated. In another comparative study, Pakfetrat *et al*. (28) compared prednisolone 5 mg/day versus colchicine (a drug that interferes with different pathways of the inflammatory process) 0.5 mg/day. Both treatments were seen to be equally effective and significantly reduced the lesion outbreaks, though colchicine produced more side effects. Thus, 5 mg/day of prednisolone seems to be a better option in reducing the signs and symptoms of the disease.

Zinc is an essential cofactor with effects upon wound reepithelization and healing that has also been investigated as a possible treatment for RAS at a dose of 150 mg, compared with dapsone (an antiinfectious sulfone used to treat leprosy and other skin conditions) at a dose of 50 mg. Both treatments were found to have important therapeutic and prophylactic properties in application to RAS, though zinc sulfate produced much faster and sustained effects (29).

Clofazimine is an antimicrobial used for the treatment of leprosy is combination with other drugs such as rifampicin and dapsone. In application to severe RAS, and when administered at a dose of 100 mg/day during 6 months, the drug was found to avoid the appearance of new lesions during the mentioned treatment period (27).

Scully *et al*. (12) recommend the use of pentoxifylline, and inhibitor of tumor necrosis factor-alpha (TNF-α), and of neutrophil function and chemotaxis, at a dose of 400 mg three times a day during one month to obtain beneficial effects in patients with RAS. However, the authors underscore that the drug does not avoid the appearance of new outbreaks and has numerous adverse effects (particularly of a gastrointestinal nature). As a result, they consider that pentoxifylline should be used as a second line treatment option in patients that fail to respond to other therapies, or as a coadjuvant to other treatments.

Immune modulators may be useful as second line treatment in different oral diseases such as oral lichen planus, and particularly in recurrent aphthous stomatitis (16). In this context, thalidomide, an immune modulator widely used in RAS, is recommended at a dose of 50-100 mg/day (12). Hello *et al*. (30) conducted a retrospective cohort study of 92 patients with severe RAS who had received treatment in the form of thalidomide. The authors found that 85% of the patients (78/92) experienced complete remission of the lesions in the first 14 days. However, 84% of the subjects suffered adverse effects. Thalidomide is known to produce many adverse effects, including teratogenicity, polyneuropathy, drowsiness, constipation, increased appetite, headache, nausea and gastric pain (12,30). Another immune modulator is levamisole, which restores normal phagocytic activity among macrophages and neutrophils, and modulates T cell mediated immunity. The drug shortens the duration of the aphthae outbreaks, as well as the number, size and frequency of the lesions (12,31). At a dose of 150 mg three times a week during 6 months, the drug is safe (31), though adverse effects have also been described, including nausea, hyperosmia, dysgeusia and agranulocytosis (12,31).

Mimura *et al*. (32) compared the efficacy of four systemic treatments for severe RAS. Prednisone was administered during two weeks, starting with 0.5 mg/kg/day as a single morning dose, and followed after one week by a reduction to half the starting dose. At the same time, the patients were randomly assigned to one of the following drugs during 6 months: thalidomide (100 mg/day), dapsone (25 mg/day during 3 days, 50 mg/day during 3 days, 75 mg/day during 3 days, and a maintenance dose of 100 mg/day), colchicine (0.5 mg/day during 7 days, 1 mg/day during 7 days, and a maintenance dose of 1.5 mg/day) or pentoxifylline (400 mg 3 times a day). Thalidomide was found to be the most effective and best tolerated treatment, with complete resolution of the RAS outbreak in 87.5% of the patients. Pentoxifylline produced benefits in 60% of the cases.

Lastly, other systemic treatments have been described, including homeopathic medicines containing borax, mercurius solubilis, natrum muriaticum, phosphorus, sulfuric acid, nitric acid, arsenicum album, nux vomica and lycopodium. These substances, diluted in 100 ml of water and administered via the oral route every 12 hours during 6 days reduced the intensity of pain and the size of the ulcers. None of the subjects had to suspend the treatment because of adverse effects. However, there is still not enough evidence to either support or refute the use of homeopathic medicines as treatment for RAS (33).

## Conclusions

- How should these patients be treated in the dental clinic?

Possible underlying systemic causes must be discarded, based on a detailed clinical history, together with complementary procedures such as laboratory tests, where required.

The predisposing factors must be identified and controlled.

The severity of the outbreak must be evaluated, along with the type of aphthae and the frequency of outbreaks. Only when the outbreaks and symptoms are continuous should drug treatment be used, and starting in all cases with local drug treatment.

- What topical drugs are available and when should they be used?

Whenever drug treatment proves necessary, because the outbreaks are continuous and the pain affects patient quality of life, the first option is topical treatment. If the patient recognizes the prodromic manifestations (early symptoms) of the disease, antiinflammatory agents such as 5% amlexanox, or topical corticosteroids such as 0.1% triamcinolone acetonide, are particularly indicated in order to prevent the formation of lesions. In the case of already established aphthae, the abovementioned drugs can be used to lessen the inflammation, though in combination with antiseptics such as chlorhexidine and triclosan, antibiotics such as tetracyclines, hyaluronic acid or pomades containing natural substances such as myrtle or quercetin, among others, with a view to affording pain relief and to accelerate ulcer healing.

- What systemic drugs are available and when should they be used?

Systemic drug treatment is indicated when the outbreaks are constant and aggressive, with major aphthae, the pain is intense, and topical treatment is unable to afford symptoms relief. Different drugs can be used to control the symptoms, such as colchicine 1.5 mg/day, dapsone 50 mg/day, clofazimine 100 mg/day or pentoxifylline 400 mg 3 times a day - though the most widely used treatment consists of systemic corticosteroids such as prednisone 25 mg/day and immune modulators such as thalidomide 50-100 mg/day, affording complete or almost complete remission of the outbreaks – though the possibility of side effects must be taken into account.
